# Histone H2B Ubiquitination Promotes the Function of the Anaphase-Promoting Complex/Cyclosome in *Schizosaccharomyces pombe*

**DOI:** 10.1534/g3.114.012625

**Published:** 2014-06-19

**Authors:** Zachary C. Elmore, Janel R. Beckley, Jun-Song Chen, Kathleen L. Gould

**Affiliations:** Department of Cell and Developmental Biology, Vanderbilt University School of Medicine, Nashville, Tennessee 37232

**Keywords:** anaphase promoting complex/cyclosome (APC/C), deubiquitinating enzymes (DUBs), SAGA complex, histone H2B, ubiquitination

## Abstract

Ubiquitination and deubiquitination of proteins are reciprocal events involved in many cellular processes, including the cell cycle. During mitosis, the metaphase to anaphase transition is regulated by the ubiquitin ligase activity of the anaphase-promoting complex/cyclosome (APC/C). Although the E3 ubiquitin ligase function of the APC/C has been well characterized, it is not clear whether deubiquitinating enzymes (DUBs) play a role in reversing APC/C substrate ubiquitination. Here we performed a genetic screen to determine what DUB, if any, antagonizes the function of the APC/C in the fission yeast *Schizosaccharomyces pombe*. We found that deletion of *ubp8*, encoding the Spt-Ada-Gcn5-Acetyl transferase (SAGA) complex associated DUB, suppressed temperature-sensitive phenotypes of APC/C mutants *cut9-665*, *lid1-6*, *cut4-533*, and *slp1-362*. Our analysis revealed that Ubp8 antagonizes APC/C function in a mechanism independent of the spindle assembly checkpoint and proteasome activity. Notably, suppression of APC/C mutants was linked to loss of Ubp8 catalytic activity and required histone H2B ubiquitination. On the basis of these data, we conclude that Ubp8 antagonizes APC/C function indirectly by modulating H2B ubiquitination status.

The purpose of cell division is to accurately replicate the genetic material of a dividing cell and evenly distribute it between mother and daughter cells. Precise degradation of critical cell-cycle regulators by the ubiquitin proteasome system (UPS) ensures unidirectionality of cell-cycle progression (reviewed in Mocciaro and Rape 2012; Wickliffe *et al.* 2009).

Targeting of cellular proteins for degradation by the UPS involves the covalent attachment of ubiquitin to substrate proteins, creating a degradation signal that targets substrates to the 26S proteasome. Ubiquitin is attached to substrates through a tightly coordinated enzyme cascade including E1-activating, E2-conjugating, and E3-ligating enzymes (reviewed in Komander and Rape 2012; Teixeira and Reed 2013; Tomko and Hochstrasser 2013).

In *Schizosaccaromyces pombe* and other eukaryotic organisms, the metaphase to anaphase transition is controlled by the anaphase-promoting complex/cyclosome (APC/C) E3 ubiquitin ligase. The APC/C carries out its mitotic function by promoting the degradation of securin and cyclin B through the UPS (reviewed in McLean *et al.* 2011; Primorac and Musacchio 2013; Teixeira and Reed 2013). To prevent precocious sister chromatid separation, the APC/C is inhibited by the spindle assembly checkpoint (SAC) (reviewed in Jia *et al.* 2013; Musacchio 2011), which is only silenced when all kinetochores achieve bipolar attachment to spindle poles. SAC inactivation promotes APC/C activation, leading to chromosome segregation and mitotic exit (reviewed in Jia *et al.* 2013; McLean *et al.* 2011; Musacchio 2011).

Ubiquitin is removed from proteins by deubiquitinating enzymes (DUBs) (reviewed in Komander *et al.* 2009; Reyes-Turcu *et al.* 2009). DUBs are cysteine or metalloproteases that are classified based on their catalytic domain structure. The 5 DUB families include ubiquitin C-terminal hydrolases, ubiquitin-specific proteases (USPs), Machado-Joseph disease proteases, JAB1/MPN/Mov34 metalloenzymes, and ovarian tumor proteases (OTU) (Nijman *et al.* 2005). DUBs have diverse roles in regulating the ubiquitin cycle. They are responsible for processing ubiquitin precursors into their conjugation competent form, cleaving ubiquitin from target proteins, trimming of ubiquitin chains, and replenishing the free ubiquitin pool (Komander *et al.* 2009; Nijman *et al.* 2005; Reyes-Turcu *et al.* 2009). In mammalian cells, the DUB USP44 reverses APC/C mediated ubiquitination of the APC/C activator Cdc20 to maintain the SAC (Stegmeier *et al.* 2007). Although it seems plausible that reversal of APC/C ubiquitination is a conserved mechanism, there is no known USP44 homolog in non-mammals.

In this work, we used a genetic approach to determine whether a conserved DUB exists that antagonizes the function of the APC/C in *S. pombe*, a model organism with many mechanisms of conserved cell cycle control. In contrast to mammals, which encode approximately 80 DUB genes (reviewed in Komander *et al.* 2009; Reyes-Turcu *et al.* 2009), the *S. pombe* genome encodes only 20 catalytically active DUBs belonging to four of the five DUB subfamilies (ubiquitin C-terminal hydrolase, USP, OTU, and JAB1/MPN/Mov34 metalloenzymes) (Kouranti *et al.* 2010). All *S. pombe* DUBs except for the proteasomal DUB Rpn11 are nonessential for viability (Iwaki *et al.* 2007; Kim *et al.* 2010; Shimanuki *et al.* 1995; Stone *et al.* 2004; Zhou *et al.* 2003) making our genetic screen straightforward. Here, we provide evidence that a single DUB, Ubp8, antagonizes the APC/C in a mechanism independent of the SAC. Genetic analysis revealed that Ubp8’s ability to antagonize APC/C function depends on its catalytic activity and the ubiquitination status of histone H2B. Accordingly, our work reveals a new interaction between chromatin signatures and cell cycle progression, mediated by a DUB.

## Materials and Methods

### Yeast strains, media, and genetic methods

*S. pombe* strains used in this study (Supporting Information, Table S1) were grown in yeast extract (YE) medium (Moreno *et al.* 1991). Crosses were performed in glutamate medium and strains were constructed by tetrad analysis. YE G418 (100 mg/L; Sigma-Aldrich, St. Louis, MO) was used for selecting Kan^R^ cells. For serial dilution spot tests, cells were cultured in liquid YE at 25°, three serial 10-fold dilutions starting at 4 × 10^6^ cells/mL were made, 4 μL of each dilution was spotted on YE plates and cells were grown at the indicated temperatures for 3–4 d. Overexpression of pREP1-His-biotin-his (HBH)-tagged ubiquitin was achieved by growth in the absence of thiamine for 18-22 hr, whereas repression was achieved by growth in the presence of 5 μg/mL of thiamine. *htb1-Flag and htb1-K119R-Flag* strains were a gift from Dr. Jason Tanny (McGill University).

### Molecular biology methods

*ubp8* was tagged after the stop codon of its endogenous open reading frame (ORF) with sequences encoding the Kanamycin resistance gene by a polymerase chain reaction (PCR)-mediated strategy (Bahler *et al.* 1998) using a lithium acetate–based transformation procedure (Keeney and Boeke 1994). Proper integration of the epitope cassette was confirmed by whole-cell PCR.

All plasmids were generated by standard molecular biology techniques. The *ubp8* gene including 500 bp upstream and downstream of the open reading frame was amplified by PCR and ligated into a PCR-Blunt vector (Life Technologies) and then subcloned into a pIRT2 vector (Hindley *et al.* 1987). *ubp8-C154S H387A* was created by mutagenizing a pIRT2-plasmid containing *ubp8^+^* using a QuikChange site-directed mutagenesis kit (Agilent Technologies). For *ubp8* gene replacements, a haploid *ubp8*::*ura4*^+^ strain was transformed with a linear *ubp8* gene fragment (digested from pIRT2- *ubp8-C154S H387A* plasmid) using standard lithium acetate transformations. Integrants were selected based on resistance to 5-FOA and validated by colony PCR using primers homologous to endogenous sequences that flank the genomic clone within pIRT2 in combination with those within the ORF. All constructs were sequenced to ensure their accuracy.

### *S. pombe* protein methods

Cell pellets were frozen in a dry ice/ethanol bath and lysed by bead disruption in NP-40 lysis buffer under denaturing sodium dodecyl sulfate (SDS) lysis conditions as previously described (Gould *et al.* 1991), except with the addition of a complete protease inhibitor mixture (Calbiochem). Cell pellets for Htb1-FLAG immunoblots were lysed by bead disruption using a FastPrep cell homogenizer (MP Biomedicals). Proteins were immunoprecipitated with IgG sepharose beads (GE Healthcare) as described previously (Kouranti *et al.* 2010). Proteins were separated on a 4–12% Bis-Tris gel (Life Technologies), transferred to Immobilon-P PVDF (Millipore) membrane, and immunoblotted with anti-FLAG (Sigma-Aldrich), anti-GFP (Roche), IgG primary and fluorescent mouse, and rabbit secondary antibodies (LI-COR Biosciences) according to the manufacturer’s instructions. H2B ubiquitination was quantified relative to total H2B protein using Odyssey software (LI-COR Biosciences).

### *In vivo* ubiquitinome purifications

HBH-tagged ubiquitin was overexpressed in *wildtype* and *ubp8∆* strains utilizing the thiamine repressible *nmt1* promoter in pREP1 (Kouranti *et al.* 2010; Maundrell 1993). Ubiquitinated proteins were purified using two-step affinity purifications performed under denaturing conditions as described (Tagwerker *et al.* 2006). In summary, cell pellets were lysed by bead disruption in buffer 1 (8 M urea, 300 mM NaCl, 50 mM NaPO4, 0.5% NP40, and 4 mM Imidazole, pH 8) and incubated with Ni^2+^-NTA agarose beads (QIAGEN) for 3–4 hr at room temperature. After incubation, beads were washed 4 times with buffer 3 (8 M urea, 300 mM NaCl, 50 mM NaPO_4_, 0.5% NP40, and 20 mM Imidazole, pH 6.3) and eluted in buffer 4 (8 M urea, 200 mM NaCl, 50 mM NaPO_4_, 0.5% NP40 and 2% SDS, 100 mM Tris and 10 mM EDTA, pH 4.3). The pH of the eluate was adjusted to 8 and streptavidin ultra-link resin (Pierce) was added and incubated overnight at room temperature. After the overnight incubation, streptavidin beads were washed 4 times with buffer 6 (8 M urea, 200 mM NaCl, 2% SDS and 100 mM Tris, pH 8) and once with buffer 7 (8 M urea, 200 mM NaCl and 100 mM Tris, pH 8). Purifications were performed in duplicate and purified proteins were subjected to mass spectrometric (MS) analysis.

### Mass spectrometry methods

Purified ubiquitin-HBH on streptavidin beads was washed three times with Tris-urea buffer (100mM Tris, pH 8.5, 8M urea). Proteins were reduced with 3mM TCEP (Tris(2-carboxyethyl)phosphine hydrochloride), alkylated with 10mM iodoacetamide, and digested with trypsin (0.4 μg of Trypsin Gold, Promega). Two-dimensional liquid chromatography–mass spectrometry 2D-LC-MS/MS analysis was performed in the following manner. Peptides were loaded onto 26-cm columns with a bomb pressure cell and then separated and analyzed by three-phase multidimensional protein identification technology on a Velos LTQ mass spectrometer (Thermo Scientific, West Palm Beach, FL) coupled to a nanoHPLC (NanoAcquity; Waters Corporation). The NanoAcquity autosampler was used for the 12 salt elution steps, each with 2 μL of ammonium acetate. Each injection was followed by elution of peptides with a 0–40% acetonitrile gradient (60 min) except the first and last injections, in which a 0–90% acetonitrile gradient was used. One full precursor MS scan (400–2000 mass-to-charge ratio) and five tandem MS scans of the most abundant ions detected in the precursor MS scan under dynamic exclusion was performed. Ions with a neutral loss of 98 Da (singly charged), 49 Da (doubly charged), or 32.7 Da (triply charged) from the parent ions during MS^2^ were subjected to MS^3^ fragmentation. MS data analysis was done as previously described (Chen *et al.* 2013) with the following changes. A newer version of Scaffold (Scaffold v4.2.0) was used and the filtering criteria were changed to: minimum of 90.0% peptide identification probability, minimum of 99.0% protein identification probability, and minimum of 2 unique peptides.

## Results

### Ubp8 antagonizes the function of the APC/C in *S. pombe*

**Figure 1:**
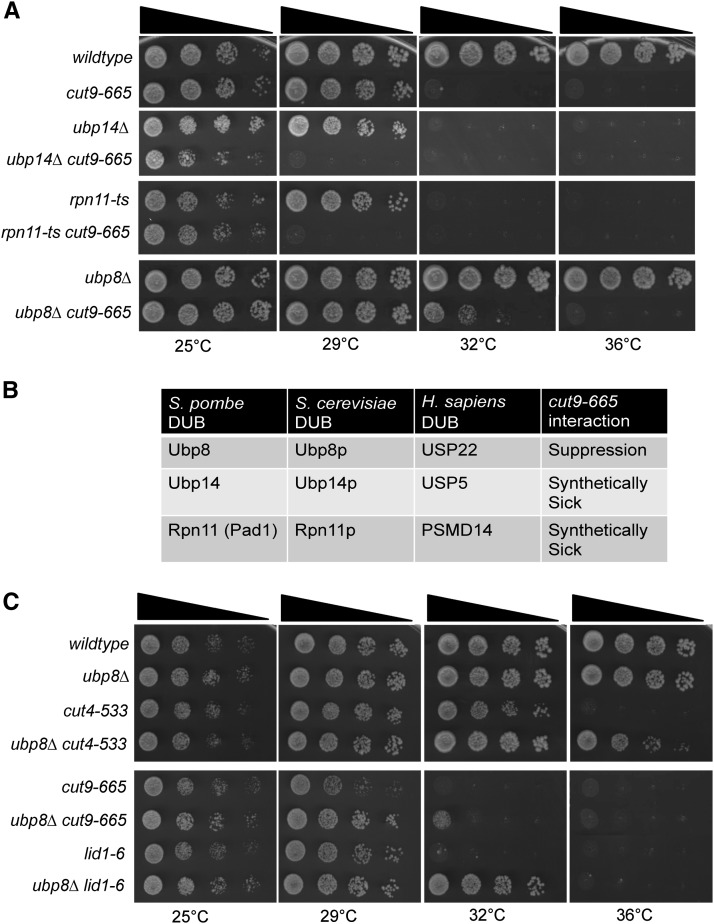
*Ubp8* antagonizes anaphase-promoting complex/cyclosome (APC/C) function in *S. pombe*. (A) *ubp8∆* suppresses the temperature-sensitive growth of *cut9-665*. Strains were grown at 25°C to an OD_595_ = 0.2. Serial dilutions (10-fold) of the indicated single and double mutant strains were spotted on yeast extract plates and incubated at the indicated temperatures. (B) List of the *S. pombe* deubiquitinating enzymes that showed genetic interactions with *cut9-665* and their homologs. (C) *ubp8∆* suppresses the temperature-sensitive phenotype of APC/C mutants *cut4-533* and *lid1-6*. Strains were grown and spotted as described in (A).

We surmised that if a DUB antagonizes APC/C function in *S. pombe*, the deletion of that DUB should suppress a mutant defective in APC/C activity. Thus, we crossed each of the 19 nonessential DUB deletion mutants and a temperature-sensitive mutant of *rpn11* (the essential DUB) to *cut9-665*, an APC/C mutant (Kouranti *et al.* 2010; Penney *et al.* 1998; Samejima and Yanagida 1994). Only three mutations affected growth of *cut9-665* (Figure 1, A and B). In a serial dilution growth test, only *ubp8∆* suppressed *cut9-665* at its semipermissive temperature (32°) (Figure 1A). *cut9-665* showed a negative genetic interaction with *ubp14∆* and *pad1-1* (a temperature-sensitive allele of the proteasomal DUB *rpn11*) (Figure 1, A and B). These DUB mutants decrease the cellular ubiquitin pool most likely contributing to the exacerbated APC/C temperature-sensitive phenotype (Penney *et al.* 1998; Verma *et al.* 2002) (see discussion).

*lid1+ and cut4+* encode other components of the APC/C whereas *slp1+* encodes the APC/C coactivator termed Cdc20p in other organisms (Yu 2007). Temperature-sensitive *lid1-6*, *cut4-533*, *cut9-665*, and *slp1-362* mutants all display a “cut” phenotype at restrictive temperatures where chromosome segregation and spindle elongation fail to occur; therefore, subsequent cytokinesis bisects the nucleus or results in segregation of DNA to only one daughter cell (Berry *et al.* 1999; Matsumoto 1997; Yamashita *et al.* 1996). To determine whether suppression of *cut9-665* by *ubp8∆* was indicative of general suppression of hypomorphic APC/C function, we tested other APC/C mutants and found that *ubp8∆* suppressed *lid1-6*, *cut4-533*, and *slp1-362* (Figure 1C, data not shown). On the basis of these data, we conclude that Ubp8 antagonizes APC/C function.

### Suppression of APC/C temperature-sensitive mutants is not dependent on the SAC or enhanced proteasome function

**Figure 2:**
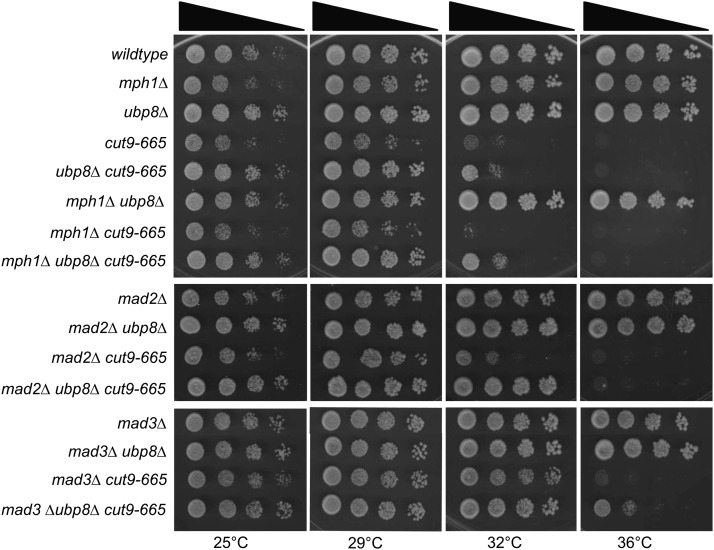
*ubp8∆* suppression of *cut9-665* is independent of the spindle assembly checkpoint. *ubp8∆* suppresses the temperature-sensitive phenotype of *cut9-665* in both *mph1^+^* and *mph1∆* strains. *ubp8∆* suppression of *cut9-665* is enhanced in *mad2∆* and *mad3∆* strains. Serial dilutions (10-fold) of the indicated single, double and triple mutant strains were spotted on yeast extract plates and incubated at the indicated temperatures.

The SAC is a well-characterized APC/C inhibitor (Jia *et al.* 2013; Musacchio 2011). Therefore, we investigated whether suppression of *cut9-665* by *ubp8∆* was achieved by diminishing SAC activity. Deletion of key SAC components *mph1∆*, *mad2∆*, and *mad3∆* (He *et al.* 1998; Sczaniecka *et al.* 2008) suppressed APC/C mutations as expected but also enhanced the suppression of the APC/C by *ubp8∆* additively (Figure 2). Interestingly, the ability of SAC components to suppress APC/C mutants was rather allele-specific (Figure 2). In any case, these data indicate that Ubp8 antagonizes the APC/C through a SAC-independent mechanism(s).

The APC/C is responsible for ubiquitinating critical cell-cycle regulatory proteins, targeting them for their subsequent degradation by the 26S proteasome (reviewed in McLean *et al.* 2011; Primorac and Musacchio 2013; Teixeira and Reed 2013). Thus, it was possible that *ubp8∆* lowered the threshold for APC/C function by enhancing proteasome-mediated degradation. To test this idea, we combined *ubp8∆* with a mutation in the proteasome subunit Mts3, *mts3-1*. This mutant is defective in proteasome-mediated proteolysis and in the metaphase to anaphase transition (Gordon *et al.* 1993; Gordon *et al.* 1996; Seeger *et al.* 1996). *ubp8∆* did not suppress the temperature-sensitive growth o*f mts3-1* (Figure S1), indicating that suppression of APC/C mutants is likely not mediated by enhanced proteasome function.

### Suppression of APC/C temperature-sensitive mutants is dependent on the activity of the SAGA DUB module

**Figure 3:**
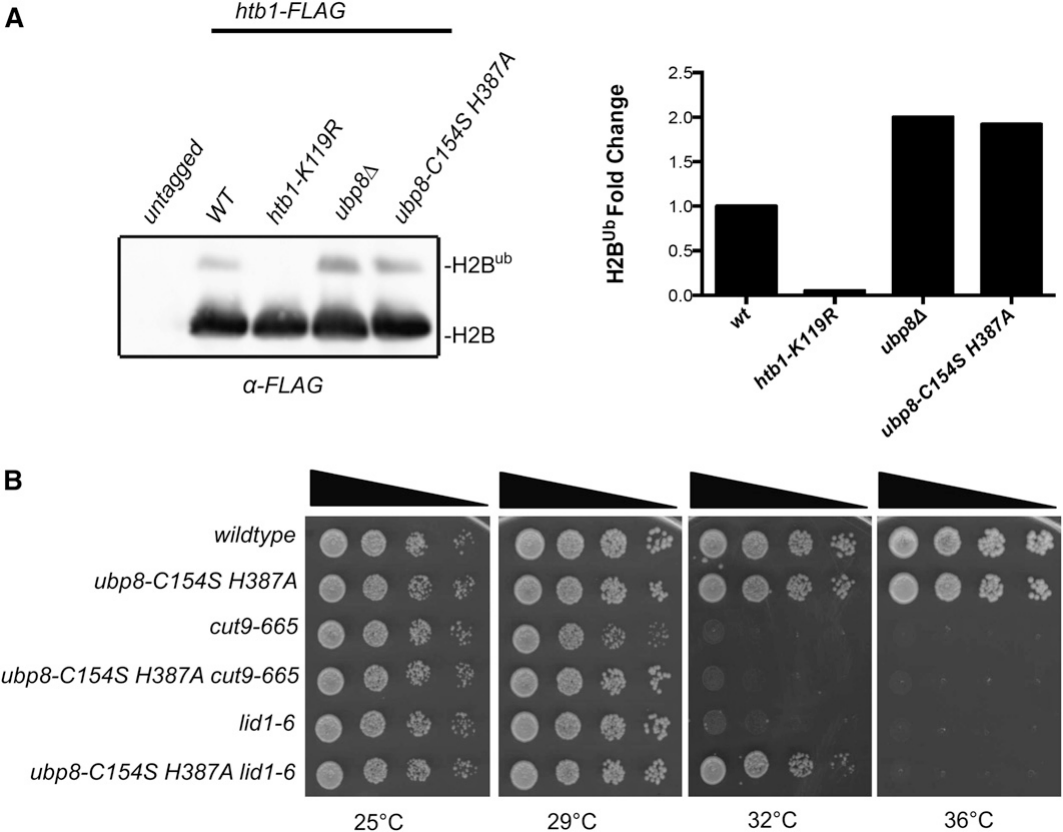
Suppression of (APC/C) temperature-sensitive mutants is dependent on the catalytic activity of Ubp8. (A) Anti-FLAG western blots of whole-cell extracts prepared from untagged and the indicated FLAG-tagged strains. H2B^ub^ corresponds to the slower migrating ubiquitinated form of H2B. Graph indicates fold change of ubiquitinated histone H2B in the indicated strain backgrounds. (B) *ubp8-C154S H387A* suppressed the temperature sensitive phenotype of *lid1-6*, and *cut9-665* mutants. Serial dilutions (10-fold) of the indicated single and double mutant strains were spotted on yeast extract plates and incubated at the indicated temperatures.

We next tested whether suppression of APC/C temperature-sensitive mutants was due to loss of Ubp8 catalytic activity or loss of the entire protein. Ubp8 is a papain-like cysteine protease that uses an Asn-His-Cys triad for catalytic function. We altered the sequence at the endogenous *ubp8* locus to produce solely Ubp8-C154S H387A, which based on sequence homology is predicted to be a catalytically inactive mutant (Ingvarsdottir *et al.* 2005). To ensure that Ubp8-C154S H387A lacked activity, we assayed the levels of ubiquitinated histone H2B, a known substrate of Ubp8 in multiple organisms (Daniel *et al.* 2004; Henry *et al.* 2003). We observed increased levels of ubiquitinated histone H2B in both *ubp8∆* and *ubp8-C154S H387A* cells (Figure 3A). Like *ubp8∆*, *ubp8-C154S H387A* suppressed the temperature sensitive phenotype of multiple APC/C mutants although it suppressed *lid1-6* the best (Figure 3B), indicating that antagonization of the APC/C by Ubp8 depends on its catalytic function.

**Figure 4:**
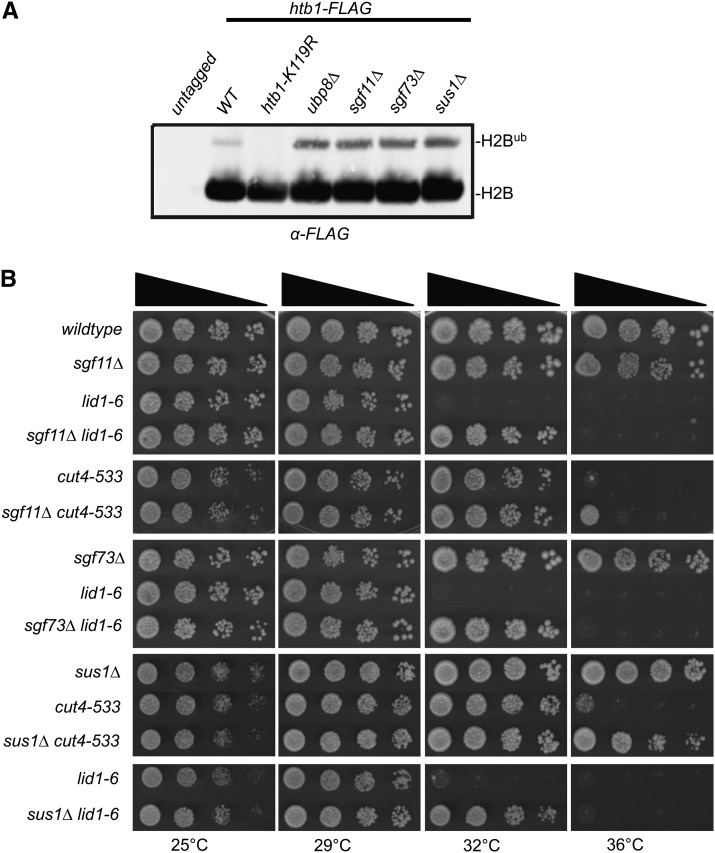
Suppression of APC/C temperature-sensitive mutants is dependent on the SAGA DUB module. (A) Anti-FLAG western blots of whole-cell extracts prepared from untagged and the indicated FLAG-tagged strains. H2B^ub^ corresponds to the slower migrating ubiquitinated form of H2B. (B) Individual deletions of SAGA DUB module components (*sgf11∆*, *sgf73∆*, and *sus1∆*) suppress the temperature-sensitive phenotype of APC/C mutants. Serial dilutions (10-fold) of the indicated single and double mutant strains were spotted on yeast extract plates and incubated at the indicated temperatures.

Ubp8 is part of the evolutionarily conserved DUB module of the SAGA transcriptional complex. In *S. pombe*, the SAGA DUB module consists of Sgf11, Sgf73, and Sus1, in addition to Ubp8 (Helmlinger *et al.* 2008). In *Saccharomyces cerevisiae*, all four analogous DUB module components are required for ubiquitin protease activity *in vitro* and *in vivo* (Lee *et al.* 2009; Weake *et al.* 2008). As expected based on these data, deletion of each *S. pombe* SAGA DUB module component (*ubp8∆*, *sgf11∆*, *sgf73∆*, *or sus1∆*) led to increased levels of ubiquitinated H2B *in vivo* (Figure 4A). Furthermore, each deletion suppressed the temperature-sensitive phenotype of APC/C mutants (Figure 4B). These results indicate that suppression of APC/C mutants is achieved by loss of the DUB activity associated with the SAGA complex.

### Suppression of APC/C temperature-sensitive mutants is specific to the SAGA DUB module

**Figure 5:**
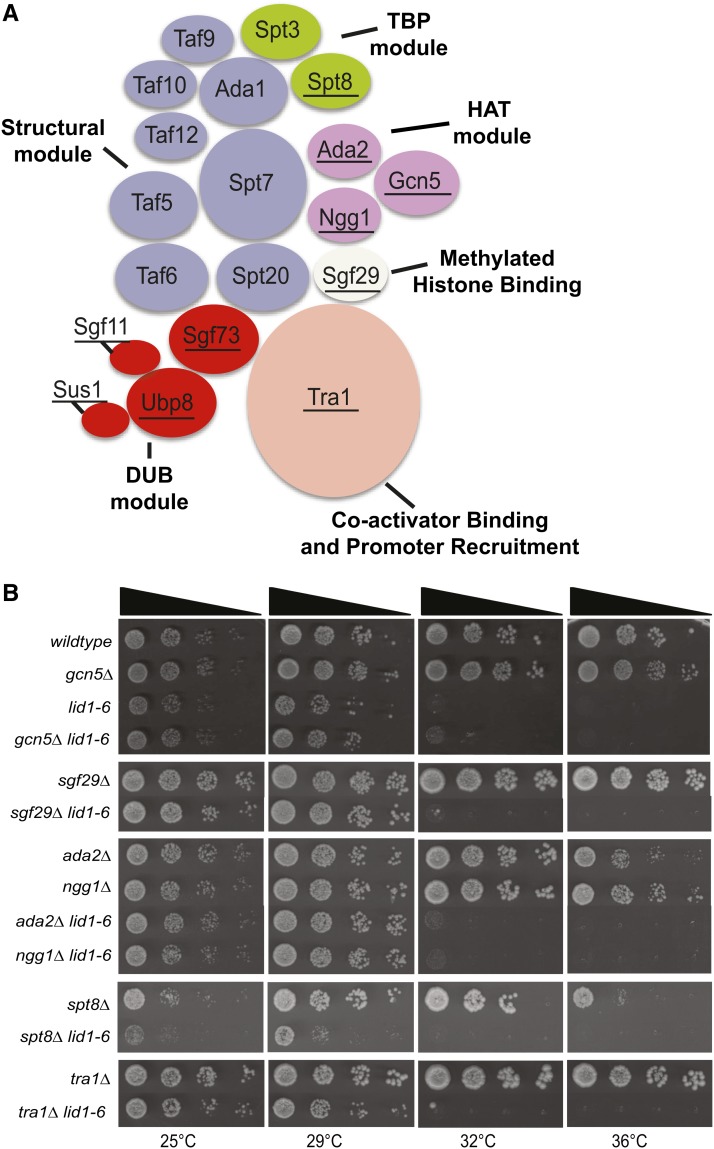
Suppression of APC/C temperature-sensitive mutants is specific to the SAGA DUB module. (A) Schematic organization of *S. pombe* SAGA complex. Structural subunits are colored lavender; Tra1 (coactivator binding and promoter recruitment), peach; histone acetyltransferase (HAT), pink; TATA-box binding protein module (TBP), green; DUB module, red; and Sgf29 (methylated histone binding), cream. Underlined subunits indicate mutants used in this study. (B) Single deletions of SAGA complex components and the effect on the temperature sensitive phenotype of *lid1-6*. Serial dilutions (10-fold) of the indicated single- and double-mutant strains were spotted on yeast extract and incubated at the indicated temperatures.

The SAGA complex is organized into distinct subcomplexes, each with discrete regulatory activities (reviewed in Koutelou *et al.* 2010; Samara and Wolberger 2011). In *S. pombe*, these modules include the DUB, histone acetyltransferase, TATA-binding protein, structural, Tra1, and Sgf29 (reviewed in Helmlinger 2012) (Figure 5A). The main function of the DUB module is to deubiquitinate histone H2B to regulate gene expression. The histone acetyltransferase module is responsible for acetylation of histone H2B and H3, whereas the TATA-binding protein module regulates preinitiation complex assembly and transcriptional activation. Tra1 has an important role in the recruitment of transcriptional coactivator complexes to specific promoters, while Sgf29 plays a role in the binding of methylated histones (Helmlinger 2012). Components of the structural module TBP-associated factors (TAFs) are responsible for maintaining SAGA architectural integrity and are involved in regulating gene expression through their association with the TFIID general transcription factor complex (Grant *et al.* 1998). In accordance with their role in multiple complexes, TAF components are encoded by essential genes in *S. pombe* (Helmlinger *et al.* 2011).

To determine whether SAGA subunits outside the DUB module affected the cells’ requirement for APC/C function, we tested whether deletion mutants of non-DUB SAGA components could suppress APC/C mutants. *gcn5∆*, *sgf29∆*, *ada2∆*, *ngg1∆*, and *tra1∆*, did not suppress the temperature-sensitive phenotype of *lid1-6* (Figure 5B). Further, *spt8∆* had a negative growth phenotype in combination with *lid1-6* (Figure 5B). These data indicate that the activity of the DUB module of the SAGA complex is responsible for antagonizing APC/C function.

### Suppression of APC/C temperature-sensitive mutants is dependent on H2B ubiquitination

To better understand how Ubp8 inhibits APC/C function, we identified putative substrates of the SAGA DUB module by semiquantitative comparison of all ubiquitinated proteins (the ubiquitinome) in wild-type cells relative to that of *ubp8∆*. We overexpressed a HBH-Ub fusion in *wildtype* and *ubp8∆* strains, performed purifications under fully denaturing conditions, and identified all ubiquitinated proteins using 2D-LC-MS/MS. We compared the abundance of each ubiquitinated protein in *ubp8∆* relative to that in the wild type to identify potential Ubp8 substrates (*i.e.*, ubiquitinated proteins enriched in the *ubp8∆* strain). Histone H2B, an important substrate of Ubp8 (reviewed in Koutelou *et al.* 2010; Samara and Wolberger 2011), was the only protein identified that was more than two-fold more abundant in *ubp8∆* than in the *wildtype* (Figure S2).

**Figure 6:**
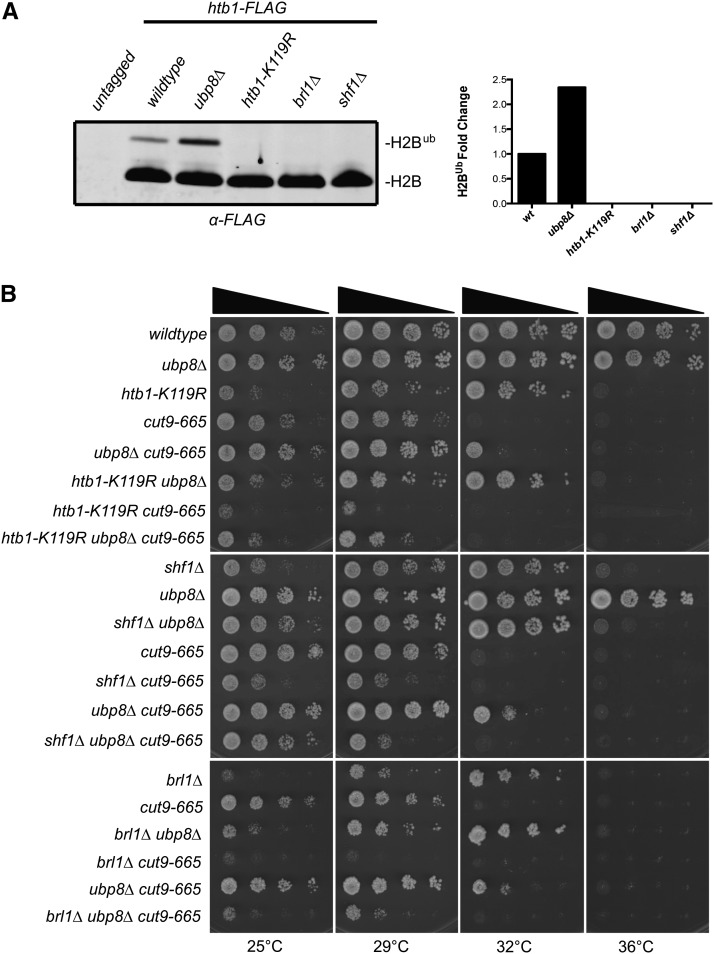
Suppression of APC/C temperature-sensitive mutants is dependent on H2B ubiquitination. (A) Anti-FLAG western blots of whole-cell extracts prepared from untagged and the indicated FLAG-tagged strains. Graph indicates fold change of ubiquitinated histone H2B in the indicated strain backgrounds. (B) Individual mutants (*brl1∆*, *htb1-K119R*, *shf1∆*) that abolish H2B ubiquitination did not suppress the temperature-sensitive phenotype of APC/C mutants when combined with *ubp8∆*. Serial dilutions of the indicated single, double, and triple mutant strains were spotted on yeast extract plates and incubated at the indicated temperatures.

With this knowledge in mind, we investigated whether the ability of *ubp8∆* to suppress APC/C mutants required histone H2B ubiquitination. BRE1-like Brl1 and small histone ubiquitination factor Shf1, components of the HULC ubiquitin ligase complex, are required in *S. pombe* for H2B ubiquitination (Tanny *et al.* 2007; Zofall and Grewal 2007). Deletions of *brl1* and *shf1* (*brl1∆* and *shf1∆*) abolished ubiquitination of histone H2B as did a lysine to arginine mutant of histone H2B in which the ubiquitin acceptor site is lost (*htb1-K119R*) (Figure 6A). *ubp8∆* no longer suppressed APC/C temperature mutants when combined with mutants in which H2B ubiquitination was abolished (Figure 6B). Furthermore, H2B ubiquitination mutants exhibited a negative growth phenotype when combined with *cut9-665* (Figure 6B). Together these results indicate that elevated levels of histone H2B ubiquitination reduce the requirement for APC/C function in *S. pombe*.

## Discussion

In this work we found that of all DUB single deletions, only one, *ubp8∆*, reduced the requirement for APC/C function in *S. pombe*. Our data support a model wherein Ubp8 antagonizes APC/C indirectly via modulating the ubiquitination status of H2B.

### Multiple DUBs influence APC/C function

In our genetic approach to identify DUB(s) that antagonize APC/C function, we found that *ubp14∆* and *pad1-1*, mutations in the conserved DUBs Ubp14 and Rpn11, respectively, showed negative genetic interactions with APC/C mutants, indicating that these DUBs are important for APC/C function. Ubp14 cleaves free ubiquitin chains to liberate monomeric ubiquitin, thereby replenishing the ubiquitin pool (Amerik *et al.* 1997). Deletion of *ubp14* leads to the accumulation of free ubiquitin chains that compete with other cellular proteins for proteasome binding, thereby interfering with normal proteasome function and presumably the degradation of APC/C substrates (Amerik *et al.* 1997). Similarly, Rpn11, an essential proteasomal DUB, cleaves the proximal ubiquitin molecule in a ubiquitin chain when polyubiquitinated substrates bind the proteasome (Verma *et al.* 2002). This proximal cleavage releases the ubiquitin chain from the proteasome and allows target proteins to be properly degraded. *pad1-1 (rpn11-ts)* mutants have compromised ubiquitin-dependent proteasome function and arrest at metaphase at the restrictive temperature (Penney *et al.* 1998; Verma *et al.* 2002). Therefore, through decreased proteasome function in *ubp14∆* and *pad1-1 (rpn11-ts)* mutants, we predict that the degradation of APC/C substrates is compromised, further exacerbating the *cut9-665* temperature sensitive phenotype.

### Suppression of APC/C mutants depends on SAGA DUB module activity

Only mutations of the DUB module of the SAGA complex suppressed mutations in APC/C components. The yeast SAGA DUB module is a highly conserved complex in which the proteins Sgf11, Sgf73, Sus1, and Ubp8 are orthologous to the human proteins ATXN7L3, ATXN7, ENY2, and USP22, respectively (Lang *et al.* 2011; Zhao *et al.* 2008). In *S. cerevisiae*, each subunit of the Ubp8-Sgf11-Sus1-Sgf73 complex makes extensive contact with all other subunits and all four components are required for DUB activity *in vitro* and *in vivo (**Lee et al. 2009*; *Weake et al. 2008**)*. In contrast, in *Drosophila melanogaster* and in human cells, the presence of Ataxin-7 (Sgf73) is not necessary for DUB activity, and, instead, loss of Ataxin-7 results in increased deubiquitination and reduced levels of H2B ubiquitination (Mohan *et al.* 2014). This is presumably through the release of an active DUB module population no longer regulated by other components of the SAGA complex. These results reveal the complexity of regulation of H2B ubiquitination and present an interesting backdrop for understanding the role of the DUB module in regulating APC/C function in higher eukaryotes.

### Increased histone H2B ubiquitination reduces the threshold for APC/C function

Histone H2B ubiquitination regulates many cellular processes, including transcriptional activation and silencing, maintenance of chromatin structure, and DNA repair (Weake and Workman 2008), and we have now linked this modification to a function in APC/C regulation in *S. pombe*. The exact mechanism by which H2B ubiquitination promotes APC/C function remains unclear. Because of its known role in transcriptional regulation, it is possible that increased H2B ubiquitination in *ubp8∆* modulates the levels of APC/C components, inhibitors or activators. However, there is no evidence in support of this explanation from the results of extensive transcriptome analyses (Helmlinger *et al.* 2011). For example, the transcript level of the *S. pombe* APC/C coactivator *slp1*, which is known to be cell cycle regulated (Anderson *et al.* 2002), is not altered in deletions of DUB module components nor are the transcripts for any core APC/C component. Transcript levels of members of the PKA pathway, an APC/C inhibitor, are also unaltered in DUB module mutants (Helmlinger *et al.* 2011).

A second possibility for the reduced requirement of APC/C function when H2B ubiquitination levels are raised in *ubp8∆* cells could be related to the effects of H2B ubiquitination on centromere structure and function. H2B mutations that affect its structure and ubiquitination lead to centromeric defects (Maruyama *et al.* 2006). Indeed, ubiquitination of H2B is required to maintain active centromeric chromatin and therefore enhances proper kinetochore formation (Sadeghi *et al.* 2014). Improved kinetochore formation could promote spindle microtubule-kinetochore attachment thereby reducing SAC activity, which could then lead to suppression of APC/C mutants. Although this is an attractive possibility, we did not obtain evidence supporting this mechanism. Importantly, we found that *ubp8∆* suppressed APC/C mutants independently of the SAC. Furthermore, *ubp8∆* was unable to suppress mutants of the essential kinetochore protein Nuf2 (*nuf2-1*, *nuf2-2*, and *nuf2-3*) (Nabetani *et al.* 2001) (Figure S3). However, more complicated mechanisms of influencing the requirement for APC/C function through an effect of H2B ubiquitination on centromeric chromatin structure cannot be ruled out.

A third possibility is that H2B ubiquitination regulates a downstream signaling event that modulates APC/C activity. For example, H2B ubiquitination is required for methylation of the Dam1 kinetochore component (Latham *et al.* 2011) and has a role in regulation of DNA replication in *S. cerevisiae* (Trujillo and Osley 2012). Thus, it is possible that Ubp8 regulates signaling events beyond chromatin that antagonize APC/C function. Further work is needed to dissect the molecular mechanism by which H2B ubiquitination impacts APC/C function.

Because of its essential roles in chromosome segregation and mitotic progression, the APC/C has become a therapeutic target for the treatment of multiple neoplastic diseases (Bassermann *et al.* 2014). Interestingly, the Ubp8 homolog USP22 has been identified as a member of an 11-gene “death from cancer” signature that acts as a predictor of tumor aggressiveness, treatment resistance, and metastatic probability in cancer patients (Atanassov and Dent 2011; Glinsky 2006; Liu *et al.* 2010). Therefore, advancing our understanding of how the SAGA DUB module controls cell division may provide insights into the role of USP22 in tumor progression.

## Supplementary Material

Supporting Information
