# Supplementary material for: Histone H2B Ubiquitination Promotes the Function of the Anaphase-Promoting Complex/Cyclosome in Schizosaccharomyces pombe
Source: G3 (Bethesda). 2014 Jun 19;4(8):1529–38. doi: 10.1534/g3.114.012625 (PMC4132182; doi:10.1534/g3.114.012625)
Supplement: Supporting Information [file supp_4_8_1529__index.html]

Histone H2B Ubiquitination Promotes the Function of the Anaphase-Promoting Complex/Cyclosome in Schizosaccharomyces pombe — Supporting Information 

# Histone H2B Ubiquitination Promotes the Function of the Anaphase-Promoting Complex/Cyclosome in *Schizosaccharomyces pombe*

## Supporting Information for Elmore *et al.*, 2014

**Files in this Data Supplement:**

- Supporting Information - Figures S1-S3 and Table S1 (PDF, 354 KB)
- Figure S1 - *ubp8Δ* does not suppress the temperature sensitive phenotype of proteasome mutants. (PDF, 184 KB)
- Figure S2 - Proteomic identification of SAGA DUB module substrates. (PDF, 164 KB)
- Figure S3 - *ubp8Δ* does not suppress the temperature sensitive phenotype of nuf2 kinetochore mutants. (PDF, 180 KB)
- Table S1 - Strains used in this study. (PDF, 120 KB)
