# Supplementary material for: Histone H2B Ubiquitination Promotes the Function of the Anaphase-Promoting Complex/Cyclosome in Schizosaccharomyces pombe
Source: G3 (Bethesda). 2014 Jun 19;4(8):1529–38. doi: 10.1534/g3.114.012625 (PMC4132182; doi:10.1534/g3.114.012625)
Supplement: Supporting Information [file supp_g3.114.012625_012625SI.pdf]

G3 · Genes | Genomes | Genetics

Histone H2B ubiquitination promotes the function of the anaphase-promoting complex/cyclosome in *Schizosaccharomyces pombe*

Zachary C. Elmore[1], Janel R. Beckley[1], Jun-Song Chen[1] and Kathleen L. Gould[1]

[1]Department of Cell and Developmental Biology, Vanderbilt University School of Medicine, Nashville, TN 37232

Corresponding Author Contact information:
Kathleen L. Gould
Mailing address: B-2309 Medical Center North, 1161 21st Avenue South, Nashville, TN 37232
Phone: 615-343-9502
Fax: 615-343-0723
email: kathy.gould@vanderbilt.edu

DOI: 10.1534/g3.114.012625

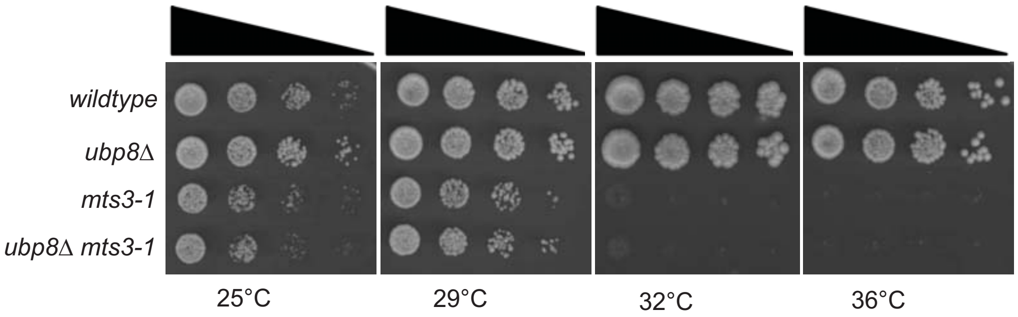

Figure S1 *ubp8Δ* does not suppress the temperature sensitive phenotype of proteasome mutants. Serial dilutions (10 fold) of the indicated single and double mutant strains were spotted on YE plates and incubated at the indicated temperatures.

ORF	Protein	Description	MW	Ratio	Normalized TSC		TSC		Coverage	
				ubp8Δ/ubp8^{+}	ubp8Δ	ubp8^{+}	ubp8Δ	ubp8^{+}	ubp8Δ	ubp8^{+}
SPBC337.08c	Ubi4	ubiquitin	43 kDa	1.0	1000	1000	1840	464	19%	27%
SPCC622.09	Htb1	histone H2B Htb1	14 kDa	3.0	176	58	324	27	52%	52%
SPAC16C9.02c		S-methyl-5-thioadenosine phosphorylase	34 kDa	1.3	3	2	5	1	8%	4%
SPCC1281.06c		acyl-coA desaturase	54 kDa	0.8	10	13	19	6	11%	11%
SPBC3H7.02		sulfate transporter	96 kDa	0.7	6	9	11	4	6%	6%
SPAC1F12.05		endocytosis regulator	42 kDa	0.6	8	13	14	6	9%	5%
SPAC18G6.14c	Rps7	40S ribosomal protein S7	22 kDa	0.6	49	86	90	40	52%	56%
SPBC1652.02		APC amino acid transporter	65 kDa	0.5	9	17	17	8	4%	4%
SPAC29B12.11c		human WW domain binding protein-2 orthol	20 kDa	0.5	2	4	4	2	10%	5%
SPBC1289.16c	Cao2	copper amine oxidase-like protein Cao2	90 kDa	0.5	1	2	2	1	5%	2%
SPCC757.03c		ThiJ domain protein	27 kDa	0.4	2	4	3	2	6%	7%
SPCP1E11.04c	Pal1	membrane associated protein Pal1	47 kDa	0.3	8	24	15	11	28%	21%
SPBC1685.13	Fhn1	Fhn1 plasma membrane organization protein	20 kDa	0.3	2	6	4	3	9%	9%
SPCC1020.10	Oca2	serine/threonine protein kinase Oca2	73 kDa	0.3	1	4	2	2	2%	4%
SPAC29B12.10c	Pgt1	glutathione transporter Pgt1	96 kDa	0.3	1	4	2	2	1%	1%
SPAC630.08c	Erg25	C-4 methylsterol oxidase	36 kDa	0.3	1	4	2	2	5%	5%
SPBC1711.04		methylenetetrahydrofolate reductase	36 kDa	0.3	1	4	2	2	8%	7%
SPBC16E9.02c		CUE domain protein	64 kDa	0.3	5	22	10	10	9%	9%
SPBC1604.21c	Ptr3	ubiquitin activating enzyme E1	113 kDa	0.3	3	11	5	5	3%	3%

Figure S2 Proteomic identification of SAGA DUB module substrates. Indicated are proteins identified from *S. pombe* ubiquitinome purifications in *wildtype* and *ubp8Δ* strains. Total spectral counts (Stone *et al.*) for each protein were normalized to the TSC for ubiquitin.

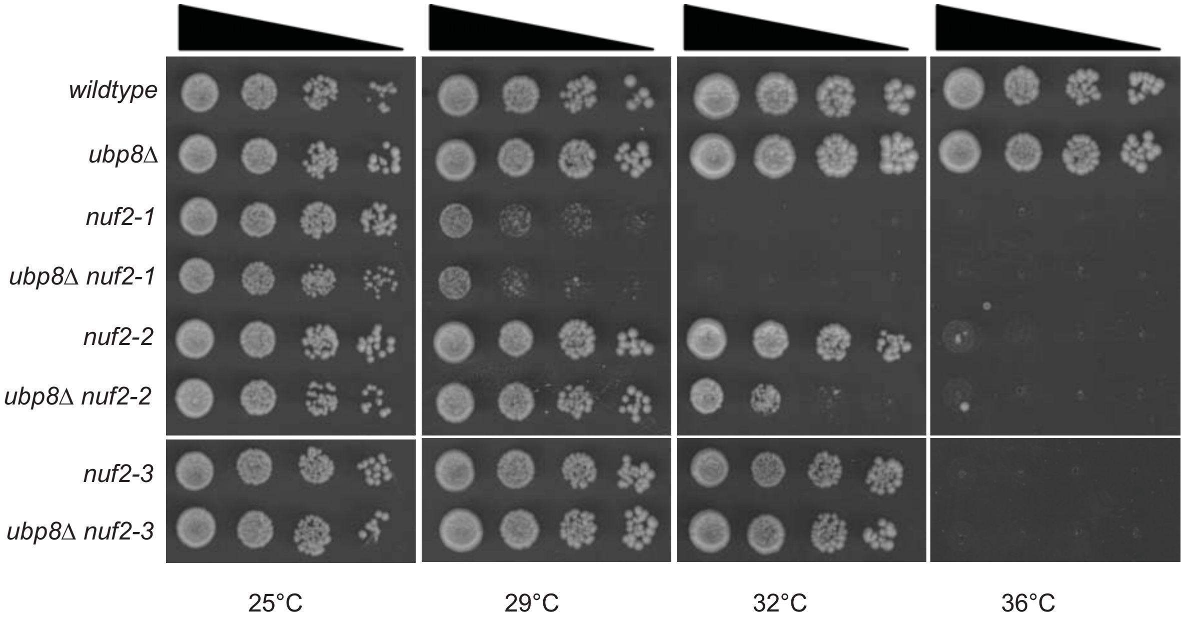

Figure S3 *ubp8Δ* does not suppress the temperature sensitive phenotype of nuf2 kinetochore mutants. Serial dilutions (10 fold) of the indicated single and double mutant strains were spotted on YE plates and incubated at the indicated temperatures.

Table S1 Strains used in this study.

Strain	Genotype	Source/reference
KGY246	*ade6*-M210 *leu1*-32 *ura4*-D18 h-	Lab stock
KGY963	*cut9-665 ade6*-M210 *leu1*-32 *ura4*-D18 h-	Lab stock
KGY1135	*mts3-1 ade6*-M21X *leu1*-32 *ura4*-D18 h-	Lab stock
KGY1150	*lid1-6 ade6-M21X leu1*-32 *ura4*-D18 h-	Lab stock
KGY2027	*cut4-533 leu1*-32 *ura4*-D18 h-	Lab stock
KGY4890	*nuf2-1*::*ura4*+ *ura4*-D18 h+	Lab stock
KGY4891	*nuf2-2*::*ura4*+ *ura4*-D18 h+	Lab stock
KGY4892	*nuf2-3*::*ura4*+ *ura4*-D18 h+	Lab stock
KGY10215	*mad2*::kan^R *ade6*-M21X *leu1*-32 *ura4*-D18 h-	Lab stock
KGY10760	*mad3*::kan^R *ade6*-M21X *leu1*-32 *ura4*-D18 h+	Lab stock
KGY11326	*pad1.1 leu1-32 h-*	Lab stock
KGY12147	*ubp14::ura4*$^+$ *leu1*-32 *ura4*-D18 h-	This study
KGY12992	*ubp8::ura4*$^+$ *ade6*-M210 *leu1*-32 *ura4*-D18 h-	Lab stock
KGY13262	*ubp14::ura4*$^+$ *ade6-M21X leu1*-32 *ura4*-D18 h-	This study
KGY13267	*ubp8::ura4*$^+$ *cut9-665 ade6*-M210 *leu1*-32 *ura4*-D18 h-	This study
KGY13754	*mph1*::kan^R *ade6*-M21X *leu1*-32 *ura4*-D18 h+	Lab stock
KGY14244	*tra1::*kan^R *ade6*-M21X *leu1*-32 *ura4*-D18 h+	Lab stock
KGY14246	*tra1::*kan^R *lid1-6 ade6*-M21X *leu1*-32 *ura4*-D18 h?	This study
KGY14251	*gcn5::*kan^R *ade6*-M21X *leu1*-32 *ura4*-D18 h+	Lab stock
KGY14252	*gcn5::*kan^R *lid1-6 ade6*-M21X *leu1*-32 *ura4*-D18 h?	This study
KGY14484	*sgf73*::kan^R *lid1-6 ade6*-M21X *leu1*-32 *ura4*-D18 h?	This study
KGY14485	*sgf29*::kan^R *ade6*-M21X *leu1*-32 *ura4*-D18 h+	Lab stock
KGY14489	*sgf73*::kan^R *cut9-665 ade6*-M21X *leu1*-32 *ura4*-D18 h?	This study
KGY14490	*sgf73*::kan^R *ade6*-M21X *leu1*-32 *ura4*-D18 h+	Lab stock
KGY14492	*spt8*::kan^R *ade6*-M21X *leu1*-32 *ura4*-D18 h+	Lab stock

KGY14493	*ada2*::kan^R *ade6*-M21X *leu1*-32 *ura4*-D18 h+	Lab stock
KGY14494	*ngg1*::kan^R *ade6*-M21X *leu1*-32 *ura4*-D18 h+	Lab stock
KGY14495	*mph1::kanR ubp8::ura4+ cut9-665 ade6-M21X leu1-32 ura4-D18 h+*	This study
KGY14614	*sus1::ura4*$^+$ *ade6*-M210 *ura4*-D18 h+	F. Winston
KGY14727	*sus1::ura4*$^+$ *lid1-6 ade6*-M210 *ura4*-D18 h?	This study
KGY14729	*sus1::ura4*$^+$ *cut4-533 ade6*-M210 *leu1*-32 *ura4*-D18 h?	This study
KGY15057	*shf1*::kan^R *ade6*-M21X *leu1*-32 *ura4*-D18 h+	Lab stock
KGY15058	*shf1::kanR cut9-665 ade6-M21X leu1-32 ura4-D18 h?*	This study
KGY15059	*shf1::kanR ubp8::ura4+ ade6-M21X leu1-32 ura4-D18 h?*	
KGY15060	*shf1::kanR ubp8::ura4+ cut9-665 ade6-M21X leu1-32 ura4-D18 h?*	This study
KGY15109	*htb1-K119R::kanR ade6-M21X leu1-32 ura4-D18 h?*	This study
KGY15110	*htb1-K119R::kanR cut9-665 ade6-M21X leu1-32 ura4-D18 h?*	This study
KGY15111	*htb1-K119R::kanR ubp8::ura4+ ade6-M21X leu1-32 ura4-D18 h?*	This study
KGY15112	*htb1-K119R::kanR ubp8::ura4+ cut9-665 ade6-M21X leu1-32 ura4-D18 h?*	This study
KGY15139	*ubp8-C154S:kanR ade6-M21X leu1-32 ura4-D18 h?*	This study
KGY15140	*ubp8-C154S:kanR lid1-6 ade6-M21X leu1-32 ura4-D18 h?*	This study
KGY15141	*ubp8-C154S:kanR cut9-665 ade6-M21X leu1-32 ura4-D18 h?*	This study
KGY15217	*htb1-FLAG:kanR ubp8-C154S H387A::kanR ade6-M21X ura4-D18 h?*	This study
KGY15253	*htb1-FLAG:kanR ade6-M21X h-*	*Tanny* et al., 2007
KGY15254	*htb1-FLAG:kanR ubp8::kanR ade6-M21X ura4-D18 h-*	This study
KGY15315	*ubp8::ura4*$^+$ *lid1-6 ade6-M21X leu1*-32 *ura4*-D18 h?+	This study
KGY15353	*brl1::kanR ade6-M21X leu1-32 ura4-D18 h+*	Lab stock
KGY15355	*ubp8::ura4*$^+$ *cut4-533 ade6-M21X leu1*-32 *ura4*-D18 h+	This study

KGY15387	*brl1::kanR htb1-FLAG:kanR ade6-M21X h?*	This study
KGY15388	*htb1-K119R-FLAG:kanR ade6-M21X h-*	*Tanny* et al., 2007
KGY15563	*brl1::kanR cut9-665 ade6-M21X leu1-32 ura4-D18 h?*	This study
KGY15564	*brl1::kanR ubp8::ura4+ cut9-665 ade6-M21X leu1-32 ura4-D18 h?*	This study
KGY15744	*shf1::kanR htb1-FLAG:kanR ade6-M21X leu1-32 ura4-D18 h?*	This study
KGY15755	*sgf11*:: kan^R *ade6*-M21X *leu1*-32 *ura4*-D18 h+	This study
KGY15783	*spt8*::kan^R *lid1-6 ade6*-M21X *leu1*-32 *ura4*-D18 h?	This study
KGY15784	*ubp8::ura4+ mts3-1 ade6-M210 leu1-32 ura4-D18 h?*	This study
KGY15800	*sgf29*::kan^R *lid1-6 ade6*-M21X *leu1*-32 *ura4*-D18 h?	This study
KGY15870	*sgf11*:: kan^R *cut4-533 ade6-M21X leu1*-32 *ura4*-D18 h?	This study
KGY15871	*sgf11*:: kan^R *lid1-6 ade6*-M21X *leu1*-32 *ura4*-D18 h?	This study
KGY15872	*mph1::kanR cut9-665 ade6-M21X leu1-32 ura4-D18 h+*	This study
KGY15873	*mph1::kanR ubp8::ura4+ ade6-M21X leu1-32 ura4-D18 h-*	This study
KGY15874	*mad3::kanR ubp8::ura4+ ade6-M21X leu1-32 ura4-D18 h?*	This study
KGY15875	*mad3::kanR cut9-665 ade6-M21X leu1-32 ura4-D18 h+*	This study
KGY15876	*mad3::kanR ubp8::ura4+ cut9-665 ade6-M21X leu1-32 ura4-D18 h-*	This study
KGY15877	*mad2::kanR ubp8::ura4+ ade6-M21X leu1-32 ura4-D18 h+*	This study
KGY15878	*mad2::kanR cut9-665 ade6-M21X leu1-32 ura4-D18 h-*	This study
KGY15879	*mad2::kanR ubp8::ura4+ cut9-665 ade6-M21X leu1-32 ura4-D18 h+*	This study
KGY15944	*pad1.1 cut9-665 ade6-M21X leu1*-32 *ura4*-D18 h+	This study
KGY15978	*sgf11::kan^R htb1-FLAG:*kan^R *ade6*-M21X h?	This study
KGY15979	*sgf73::kan^R htb1-FLAG:*kan^R *ade6*-M21X h?	This study
KGY15980	*sus1::ura4$^+$ htb1-FLAG:*kan^R *ura4*-D18 *ade6*-M21X h?	This study
KGY16395	*ada2*::kan^R *lid1-6 ade6*-M21X *leu1*-32 *ura4*-D18 h?	This study
KGY16396	*ngg1*::kan^R *lid1-6 ade6*-M21X *leu1*-32 *ura4*-D18 h?	This study

KGY16557	*ubp8::ura4+ nuf2-1::ura4+ ura4-D18 h?*	This study
KGY16558	*ubp8::ura4+ nuf2-2::ura4+ ura4-D18 h?*	This study
KGY16559	*ubp8::ura4+ nuf2-3::ura4+ ura4-D18 h?*	This study
