# Supplementary material for: Histone H2B Ubiquitination Promotes the Function of the Anaphase-Promoting Complex/Cyclosome in Schizosaccharomyces pombe
Source: G3 (Bethesda). 2014 Jun 19;4(8):1529–38. doi: 10.1534/g3.114.012625 (PMC4132182; doi:10.1534/g3.114.012625)
Supplement: Supporting Information [file supp_g3.114.012625_FigureS1.pdf]

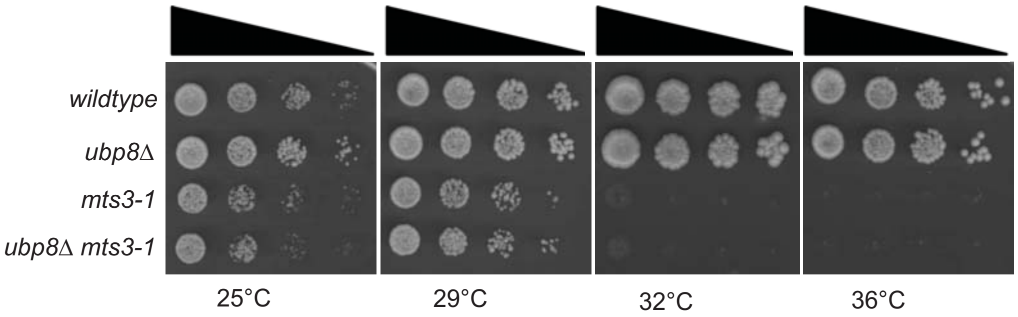

Figure S1 *ubp8Δ* does not suppress the temperature sensitive phenotype of proteasome mutants. Serial dilutions (10 fold) of the indicated single and double mutant strains were spotted on YE plates and incubated at the indicated temperatures.
