# Supplementary material for: Histone H2B Ubiquitination Promotes the Function of the Anaphase-Promoting Complex/Cyclosome in Schizosaccharomyces pombe
Source: G3 (Bethesda). 2014 Jun 19;4(8):1529–38. doi: 10.1534/g3.114.012625 (PMC4132182; doi:10.1534/g3.114.012625)
Supplement: Supporting Information [file supp_g3.114.012625_FigureS2.pdf]

ORF	Protein	Description	MW	Ratio	Normalized TSC		TSC		Coverage	
				ubp8Δ/ubp8⁺	ubp8Δ	ubp8⁺	ubp8Δ	ubp8⁺	ubp8Δ	ubp8⁺
SPBC337.08c	Ubi4	ubiquitin	43 kDa	1.0	1000	1000	1840	464	19%	27%
SPCC622.09	Htb1	histone H2B Htb1	14 kDa	3.0	176	58	324	27	52%	52%
SPAC16C9.02c		S-methyl-5-thioadenosine phosphorylase	34 kDa	1.3	3	2	5	1	8%	4%
SPCC1281.06c		acyl-coA desaturase	54 kDa	0.8	10	13	19	6	11%	11%
SPBC3H7.02		sulfate transporter	96 kDa	0.7	6	9	11	4	6%	6%
SPAC1F12.05		endocytosis regulator	42 kDa	0.6	8	13	14	6	9%	5%
SPAC18G6.14c	Rps7	40S ribosomal protein S7	22 kDa	0.6	49	86	90	40	52%	56%
SPBC1652.02		APC amino acid transporter	65 kDa	0.5	9	17	17	8	4%	4%
SPAC29B12.11c		human WW domain binding protein-2 orthol	20 kDa	0.5	2	4	4	2	10%	5%
SPBC1289.16c	Cao2	copper amine oxidase-like protein Cao2	90 kDa	0.5	1	2	2	1	5%	2%
SPCC757.03c		ThiJ domain protein	27 kDa	0.4	2	4	3	2	6%	7%
SPCP1E11.04c	Pal1	membrane associated protein Pal1	47 kDa	0.3	8	24	15	11	28%	21%
SPBC1685.13	Fhn1	Fhn1 plasma membrane organization protein	20 kDa	0.3	2	6	4	3	9%	9%
SPCC1020.10	Oca2	serine/threonine protein kinase Oca2	73 kDa	0.3	1	4	2	2	2%	4%
SPAC29B12.10c	Pgt1	glutathione transporter Pgt1	96 kDa	0.3	1	4	2	2	1%	1%
SPAC630.08c	Erg25	C-4 methylsterol oxidase	36 kDa	0.3	1	4	2	2	5%	5%
SPBC1711.04		methylenetetrahydrofolate reductase	36 kDa	0.3	1	4	2	2	8%	7%
SPBC16E9.02c		CUE domain protein	64 kDa	0.3	5	22	10	10	9%	9%
SPBC1604.21c	Ptr3	ubiquitin activating enzyme E1	113 kDa	0.3	3	11	5	5	3%	3%

Figure S2 Proteomic identification of SAGA DUB module substrates. Indicated are proteins identified from *S. pombe* ubiquitinome purifications in *wildtype* and *ubp8Δ* strains. Total spectral counts (Stone *et al.*) for each protein were normalized to the TSC for ubiquitin.
