# Supplementary material for: Histone H2B Ubiquitination Promotes the Function of the Anaphase-Promoting Complex/Cyclosome in Schizosaccharomyces pombe
Source: G3 (Bethesda). 2014 Jun 19;4(8):1529–38. doi: 10.1534/g3.114.012625 (PMC4132182; doi:10.1534/g3.114.012625)
Supplement: Supporting Information [file supp_g3.114.012625_TableS1.pdf]

Table S1 Strains used in this study.

Strain	Genotype	Source/reference
KGY246	*ade6*-M210 *leu1*-32 *ura4*-D18 h-	Lab stock
KGY963	*cut9-665 ade6*-M210 *leu1*-32 *ura4*-D18 h-	Lab stock
KGY1135	*mts3-1 ade6*-M21X *leu1*-32 *ura4*-D18 h-	Lab stock
KGY1150	*lid1-6 ade6-M21X leu1*-32 *ura4*-D18 h-	Lab stock
KGY2027	*cut4-533 leu1*-32 *ura4*-D18 h-	Lab stock
KGY4890	*nuf2-1*::*ura4*+ *ura4*-D18 h+	Lab stock
KGY4891	*nuf2-2*::*ura4*+ *ura4*-D18 h+	Lab stock
KGY4892	*nuf2-3*::*ura4*+ *ura4*-D18 h+	Lab stock
KGY10215	*mad2*::kan^R *ade6*-M21X *leu1*-32 *ura4*-D18 h-	Lab stock
KGY10760	*mad3*::kan^R *ade6*-M21X *leu1*-32 *ura4*-D18 h+	Lab stock
KGY11326	*pad1.1 leu1-32 h-*	Lab stock
KGY12147	*ubp14::ura4*$^+$ *leu1*-32 *ura4*-D18 h-	This study
KGY12992	*ubp8::ura4*$^+$ *ade6*-M210 *leu1*-32 *ura4*-D18 h-	Lab stock
KGY13262	*ubp14::ura4*$^+$ *ade6-M21X leu1*-32 *ura4*-D18 h-	This study
KGY13267	*ubp8::ura4*$^+$ *cut9-665 ade6*-M210 *leu1*-32 *ura4*-D18 h-	This study
KGY13754	*mph1*::kan^R *ade6*-M21X *leu1*-32 *ura4*-D18 h+	Lab stock
KGY14244	*tra1::*kan^R *ade6*-M21X *leu1*-32 *ura4*-D18 h+	Lab stock
KGY14246	*tra1::*kan^R *lid1-6 ade6*-M21X *leu1*-32 *ura4*-D18 h?	This study
KGY14251	*gcn5::*kan^R *ade6*-M21X *leu1*-32 *ura4*-D18 h+	Lab stock
KGY14252	*gcn5::*kan^R *lid1-6 ade6*-M21X *leu1*-32 *ura4*-D18 h?	This study
KGY14484	*sgf73*::kan^R *lid1-6 ade6*-M21X *leu1*-32 *ura4*-D18 h?	This study
KGY14485	*sgf29*::kan^R *ade6*-M21X *leu1*-32 *ura4*-D18 h+	Lab stock
KGY14489	*sgf73*::kan^R *cut9-665 ade6*-M21X *leu1*-32 *ura4*-D18 h?	This study
KGY14490	*sgf73*::kan^R *ade6*-M21X *leu1*-32 *ura4*-D18 h+	Lab stock
KGY14492	*spt8*::kan^R *ade6*-M21X *leu1*-32 *ura4*-D18 h+	Lab stock

KGY14493	*ada2*::kan^R *ade6*-M21X *leu1*-32 *ura4*-D18 h+	Lab stock
KGY14494	*ngg1*::kan^R *ade6*-M21X *leu1*-32 *ura4*-D18 h+	Lab stock
KGY14495	*mph1::kanR ubp8::ura4+ cut9-665 ade6-M21X leu1-32 ura4-D18 h+*	This study
KGY14614	*sus1::ura4*$^+$ *ade6*-M210 *ura4*-D18 h+	F. Winston
KGY14727	*sus1::ura4*$^+$ *lid1-6 ade6*-M210 *ura4*-D18 h?	This study
KGY14729	*sus1::ura4*$^+$ *cut4-533 ade6*-M210 *leu1*-32 *ura4*-D18 h?	This study
KGY15057	*shf1*::kan^R *ade6*-M21X *leu1*-32 *ura4*-D18 h+	Lab stock
KGY15058	*shf1::kanR cut9-665 ade6-M21X leu1-32 ura4-D18 h?*	This study
KGY15059	*shf1::kanR ubp8::ura4+ ade6-M21X leu1-32 ura4-D18 h?*	
KGY15060	*shf1::kanR ubp8::ura4+ cut9-665 ade6-M21X leu1-32 ura4-D18 h?*	This study
KGY15109	*htb1-K119R::kanR ade6-M21X leu1-32 ura4-D18 h?*	This study
KGY15110	*htb1-K119R::kanR cut9-665 ade6-M21X leu1-32 ura4-D18 h?*	This study
KGY15111	*htb1-K119R::kanR ubp8::ura4+ ade6-M21X leu1-32 ura4-D18 h?*	This study
KGY15112	*htb1-K119R::kanR ubp8::ura4+ cut9-665 ade6-M21X leu1-32 ura4-D18 h?*	This study
KGY15139	*ubp8-C154S:kanR ade6-M21X leu1-32 ura4-D18 h?*	This study
KGY15140	*ubp8-C154S:kanR lid1-6 ade6-M21X leu1-32 ura4-D18 h?*	This study
KGY15141	*ubp8-C154S:kanR cut9-665 ade6-M21X leu1-32 ura4-D18 h?*	This study
KGY15217	*htb1-FLAG:kanR ubp8-C154S H387A::kanR ade6-M21X ura4-D18 h?*	This study
KGY15253	*htb1-FLAG:kanR ade6-M21X h-*	*Tanny* et al., 2007
KGY15254	*htb1-FLAG:kanR ubp8::kanR ade6-M21X ura4-D18 h-*	This study
KGY15315	*ubp8::ura4*$^+$ *lid1-6 ade6-M21X leu1*-32 *ura4*-D18 h?+	This study
KGY15353	*brl1::kanR ade6-M21X leu1-32 ura4-D18 h+*	Lab stock
KGY15355	*ubp8::ura4*$^+$ *cut4-533 ade6-M21X leu1*-32 *ura4*-D18 h+	This study

KGY15387	*brl1::kanR htb1-FLAG:kanR ade6-M21X h?*	This study
KGY15388	*htb1-K119R-FLAG:kanR ade6-M21X h-*	*Tanny* et al., 2007
KGY15563	*brl1::kanR cut9-665 ade6-M21X leu1-32 ura4-D18 h?*	This study
KGY15564	*brl1::kanR ubp8::ura4+ cut9-665 ade6-M21X leu1-32 ura4-D18 h?*	This study
KGY15744	*shf1::kanR htb1-FLAG:kanR ade6-M21X leu1-32 ura4-D18 h?*	This study
KGY15755	*sgf11*:: kan^R *ade6*-M21X *leu1*-32 *ura4*-D18 h+	This study
KGY15783	*spt8*::kan^R *lid1-6 ade6*-M21X *leu1*-32 *ura4*-D18 h?	This study
KGY15784	*ubp8::ura4+ mts3-1 ade6-M210 leu1-32 ura4-D18 h?*	This study
KGY15800	*sgf29*::kan^R *lid1-6 ade6*-M21X *leu1*-32 *ura4*-D18 h?	This study
KGY15870	*sgf11*:: kan^R *cut4-533 ade6-M21X leu1*-32 *ura4*-D18 h?	This study
KGY15871	*sgf11*:: kan^R *lid1-6 ade6*-M21X *leu1*-32 *ura4*-D18 h?	This study
KGY15872	*mph1::kanR cut9-665 ade6-M21X leu1-32 ura4-D18 h+*	This study
KGY15873	*mph1::kanR ubp8::ura4+ ade6-M21X leu1-32 ura4-D18 h-*	This study
KGY15874	*mad3::kanR ubp8::ura4+ ade6-M21X leu1-32 ura4-D18 h?*	This study
KGY15875	*mad3::kanR cut9-665 ade6-M21X leu1-32 ura4-D18 h+*	This study
KGY15876	*mad3::kanR ubp8::ura4+ cut9-665 ade6-M21X leu1-32 ura4-D18 h-*	This study
KGY15877	*mad2::kanR ubp8::ura4+ ade6-M21X leu1-32 ura4-D18 h+*	This study
KGY15878	*mad2::kanR cut9-665 ade6-M21X leu1-32 ura4-D18 h-*	This study
KGY15879	*mad2::kanR ubp8::ura4+ cut9-665 ade6-M21X leu1-32 ura4-D18 h+*	This study
KGY15944	*pad1.1 cut9-665 ade6-M21X leu1*-32 *ura4*-D18 h+	This study
KGY15978	*sgf11::kan^R htb1-FLAG:*kan^R *ade6*-M21X h?	This study
KGY15979	*sgf73::kan^R htb1-FLAG:*kan^R *ade6*-M21X h?	This study
KGY15980	*sus1::ura4$^+$ htb1-FLAG:*kan^R *ura4*-D18 *ade6*-M21X h?	This study
KGY16395	*ada2*::kan^R *lid1-6 ade6*-M21X *leu1*-32 *ura4*-D18 h?	This study
KGY16396	*ngg1*::kan^R *lid1-6 ade6*-M21X *leu1*-32 *ura4*-D18 h?	This study

KGY16557	*ubp8::ura4+ nuf2-1::ura4+ ura4-D18 h?*	This study
KGY16558	*ubp8::ura4+ nuf2-2::ura4+ ura4-D18 h?*	This study
KGY16559	*ubp8::ura4+ nuf2-3::ura4+ ura4-D18 h?*	This study
